# Exploring influential factors on patient safety culture in delirium nursing care within long-term care facilities: a cross-sectional survey

**DOI:** 10.1186/s12913-023-10452-4

**Published:** 2023-12-14

**Authors:** Se Hee Kim, Kyoung Ja Moon

**Affiliations:** Keimyung University, 1095 Dalgubeol-daero, Dalse-gu, 42601 Daegu, South Korea

**Keywords:** Patient safety culture, Long-term care facility, Delirium, Nurses

## Abstract

**Background:**

Elderly residents with physical and cognitive impairments in long-term care facilities are vulnerable to safety risks.

**Purpose:**

This study investigated factors that influence patient safety cultures in delirium nursing care in long-term care facilities.

**Methods:**

A cross-sectional survey was conducted among 214 nurses working in 12 long-term care facilities using a structured questionnaire from February 15, 2022, to March 14, 2022. Data analysis was performed using Pearson’s correlation coefficients and hierarchical analysis with SPSS/WIN 25.0 software.

**Results:**

Significant factors associated with patient safety culture were identified. Organizational factors included the availability of delirium care manuals, nursing education and experience in delirium care, and the perceived necessity of delirium education. Individual factors included nurse-to-patient ratios, and nurses marital status.

**Conclusion:**

To foster a strong patient safety culture, attention should be given to the availability of delirium care resources, the promotion of specialized and ongoing education and experience, and adequate staffing levels.

## Background

Patient safety refers to the elimination of potential harm to patients and the prevention of avoidable mortality while providing medical services [1]. To improve patient safety and the quality of healthcare services, a culture of patient safety must be fostered [2]. Patient safety culture refers to the values, attitudes, and behavioral patterns of individuals and groups that determine the approach to patient safety management and competency within healthcare organizations [3]. Relevant aspects of patient safety culture include the leadership, teamwork, work environment, safety values, risk awareness, knowledge and attitudes, policies and procedures, and communication [4]. The interest and commitment of management to patient safety are important to enhance the patient safety culture in addition to implementing a reporting suitable for the work environment and designating a patient safety officer [5].

A significant number of elderly residents with both physical and cognitive impairments are in Korea, especially in long-term care facilities (LCF), making them vulnerable to patient safety risks [6]. Nurses in LCF are particularly concerned for the safety of such residents and face difficulties in providing nursing care because of the stress from excessive workloads involving certification assessment preparations, administrative work, and the supervision of nursing assistants [7]. Further, the nurse-to-patient ratio in LCF averages 1:35, rendering the provision of adequate nursing interventions challenging. The shortage of nursing staff, when compared with the nurse-to-patient ratio of 1:15 in general hospitals, [8] leads to the inability to provide essential nursing interventions, including patient safety measures [9]. Considering the unique characteristics of LCF, the assessment of patient safety culture among LCF nurses is necessary [2].

Patient safety risk factors for LCF include medication errors, hospital-acquired infections, delirium, falls, and pneumonia [10]. Among these, the prevalence of delirium in LCFs is 48‒89% [11]. Delirium is characterized by acute impairments in attention and cognition that primarily manifest as disturbances in consciousness, attention, cognition, and perception, but can also affect sleep, psychomotor activity, and emotions [11]. It typically occurs over a short period of a few hours or days and exhibits fluctuating symptoms throughout occurrence [11].

Delirium, common among elderly residents, increases mortality rates and complications, and prolongs hospitalization, leading to increased healthcare costs [12]. The early detection and intervention of delirium can reduce mortality rates and shorten the length of hospitalization, emphasizing its importance [12, 13]. Nursing care provided by nurses, who spend the most time with patients, plays a crucial role in the early detection and intervention of delirium. The prognosis of delirium-affected patients can vary depending on the quality of nursing care [14].

Most of the delirium-related research conducted in Korea has focused on critically ill patients and nurses in general hospitals [15]. Studies specifically targeting LCF nurses are scarce. While LCF nurses recognize the importance of nursing interventions such as infection control, safety management, medication management, and pressure ulcer prevention, they face difficulties in providing delirium nursing care, signifying the need for research in this regard [16]. This study investigated the factors influencing patient safety culture among nurses in LCF regarding delirium nursing care.

## Methods

### Design

This study is a cross-sectional design conducted from February 15th, 2022, to March 14th, 2022, among nurses working in 12 long-term care facilities across three cities in the Daegu-Gyeongbuk region.

### Participants

Nurses with a minimum of six months of direct nursing experience in 12 LCFs in three cities have participated. The 12 LCFs range in size from 100 to 350 beds, with the majority of patients being elderly individuals aged 65 and above. The final sample size was 175. It was calculated using G-Power 3.1.9.7, an effect size of 0.15, power of 0.80, significance level of 0.05, and 26 predictor variables based on a previous study [17]. Considering a dropout rate of 20%, a total of 219 participants were invited to participate. To adequately represent the nursing experience within long-term care facilities, individuals with less than six years of experience and nurse administrators primarily focused on nursing administration rather than direct patient care were excluded from the study.

### Variables and measurements

#### Patient safety culture

The patient safety culture tool used included K-HSOPSC Version 2.0, [18] the Korean version of AHRQ’s HSOPSC Version 2.0, [19] and a revised version of the tool [4]. K-HSOPSC Version 2.0 [18] consists of 32 items across five dimensions: work area, immediate supervisor/manager, communication, reporting of patient safety events, and hospital. The Korean Patient Safety Culture tool [4] comprises 12 items across three dimensions: patient safety knowledge/attitude, patient safety policy/procedure, and patient safety priority. This study combined and revised the two tools to form a total of 44 items across eight dimensions. Each item was rated on a 5-point Likert scale, with higher scores indicating a higher level of patient safety culture. There were 14 negative items.

The use of the combined and revised version of the K-HSOPSC and the Korean Patient Safety Culture tool [4] was to accurately reflect the reality of patient safety culture in Korean LCFs, as the Korean Institute of Hospital Accreditation utilizes the latter [4]. To modify and improve the two tools, expert validity testing was conducted with three nursing professors specializing in the relevant field and three nurses with over five years’ LCF experience. The items were evaluated using a 4-point Likert scale, and for items requiring modification, specific details were provided to incorporate the feedback and develop the final version of the tool.

The Item Level-Content Validity Index for the tool was calculated to be 1.00; Cronbach’s ⍺ was 0.71‒0.83 for K-HSOPSC Version 2.0 [18] and 0.72‒0.89 for AHRQ’s HSOPSC Version 2.0 [19]. The Korean Patient Safety Culture tool [4] had a Cronbach’s ⍺ that ranged from 0.66 to 0.81, and the Cronbach’s ⍺ for the tool utilized in this study ranged from 0.67 to 0.91.

#### Delirium nursing care

The delirium nursing care performance tool used in this study measured nurses’ delirium risk factor assessments and nursing care performance for delirium patients. The tool was adapted from the evidence-based delirium nursing care tool for cancer patients [20]. This tool consists of 25 items, with response options ranging from “always performed” (4 points), “frequently performed” (3 points), “occasionally performed” (2 points), to “rarely performed” (1 point). Higher scores indicate higher levels of delirium nursing care performance. Cronbach’s ⍺ was 0.75 for the study by Park and Gu [20] and 0.90 for this study.

### Data collection

After explaining the research purpose and objectives to the department chairs and obtaining permission for data collection, the recruitment announcements for study participants were posted on LCF bulletin boards. Participants were met in person to obtain written consent. Among the collected questionnaires, a total of 214 valid responses were analyzed after excluding five questionnaires with inadequate responses. Given the worsening of the COVID-19 pandemic during the survey period, data from five LCFs were collected using Google Forms rather than in person in accordance with government guidelines for infection control. Participants were informed that the collected data would be treated anonymously and used solely for research purposes, and that they had the right to withdraw from the study at any time. Completed surveys were placed in envelopes by the participants and collected by the researchers.

### Data analysis

The data for this study were analyzed using SPSS 25.0. Frequency analysis and descriptive statistics were used to analyze frequencies, percentages, means, and standard deviations to understand the general characteristics of the participants. Independent-sample t-tests and one-way ANOVA were conducted to examine the differences in patient safety culture and delirium nursing care performance according to the general characteristics of the participants. Post-hoc tests were performed using Scheffé’s test, and the correlation between patient safety culture and delirium nursing care performance was analyzed using Pearson’s correlation coefficient. A hierarchical model was constructed, considering the environmental characteristics of LCFs and the characteristics of nurses related to delirium. As a result of hierarchical regression analysis, the regression model was significant (F = 17.88, *P* < .001).

## Results

### General and delirium care related characteristics of participants

The general characteristics of the participants are presented in Table 1. They were predominantly female (90.7%), with 45.3% being under the age of 30 and 54.7% being 30 years or older. The majority were married (73.8%), 45.8% had a vocational bachelor’s degree, and 54.2% had a bachelor’s degree or higher. Overall, 44.4% had less than 10 years, 55.6% had 10 years or more, and 62.6% had less than five years of LCF experience in total. The most common work schedule was rotating shift work (68.2%); 77.1% responded that they had received education on delirium, and 97.7% agreed on the need for delirium education.

**Table 1:** General and Delirium Care related Characteristics of Participants (N = 214)

Variables	Categories	n(%) or M ± SD
Gender	Female	194(90.7)
Age (year)	< 30	97(45.3)
	≥ 30	117(54.7)
Marital status	Unmarried Married	56(26.2) 158(73.8)
Educational level	Associate degree	98(45.8)
	Bachelor’s degree	116(54.2)
Total clinical experience (year)	< 10	95(44.4)
	≥ 10	119(55.6)
The current LCFª tenure (year)	< 5	134(62.6)
	≥ 5	80(37.4)
The total number of LCFª beds	< 200	134(62.6)
	≥ 200	80(37.4)
Nurse-to-patient ratio	10 ~ < 30	115(53.7)
	≥ 30	99(46.3)
Total working hours (week)	< 40	95(44.4)
	≥ 40	119(55.6)
Work shifts	Shift	146(68.2)
	Non-shift	68(31.8)
Experience of education on delirium	Yes	165(77.1)
	No	49(22.9)
Pathway to nursing education on delirium	At work	29(13.6)
	At college	88(41.1)
	Others*	97(45.3)
Presence of manuals for delirium care on the ward	Yes	69(32.2)
	No	145(67.8)
Number of caring experiences with delirious patients (month)	< 5	148(69.2)
	≥ 5	66(30.8)
Necessity of education on delirium	Yes	209(97.7)
	No	5(2.3)
Perception of patient safety (self-awareness)	Poor	23(10.7)
	Acceptable	117(54.7)
	Very good	74(34.6)

ªLCF: Long-term care facility, *others: through a colleague or naturally through clinical exposure

### Scores on patient safety culture and nursing care for delirium

Patient safety culture was measured on a scale of 1‒5, with an average score of 3.47 ± 0.48. The average scores for the dimensions were 3.95 ± 0.58 for patient safety knowledge/attitude, 3.69 ± 0.63 for communication, 3.67 ± 0.65 for immediate supervisor/manager, 3.47 ± 0.74 for patient safety policy/procedure, 3.46 ± 0.66 for hospital, 3.28 ± 0.50 for unit/work area, 3.19 ± 0.79 for patient safety priority, and 3.13 ± 0.61 for reporting of patient safety events. Delirium nursing care performance was measured on a scale of 1‒4, with an average score of 2.75 ± 0.45 (Table 2).

**Table 2:** Scoring on patient safety culture and nursing care of delirium (N = 214)

Variables	Range	M ± SD	Min	Max
Patient safety culture(Over all)	1 ~ 5	3.47 ± 0.48	1.86	4.82
Patient safety knowledge and attitude	1 ~ 5	3.95 ± 0.58	2.00	5.00
Communication	1 ~ 5	3.69 ± 0.63	1.57	5.00
Supervisor, manager, or clinical leader	1 ~ 5	3.67 ± 0.65	1.67	5.00
Patient safety policy and procedure	1 ~ 5	3.47 ± 0.74	1.25	5.00
Hospital	1 ~ 5	3.46 ± 0.66	1.50	5.00
Unit/work area	1 ~ 5	3.28 ± 0.50	1.62	4.77
Patient safety priority	1 ~ 5	3.19 ± 0.79	1.00	5.00
Reporting patient safety events	1 ~ 5	3.13 ± 0.61	1.00	4.67
Nursing care for delirium	1 ~ 4	2.75 ± 0.45	1.00	3.80

### Correlations among patient safety culture and delirium nursing care

A significant positive correlation was observed between delirium nursing care performance and patient safety culture (r = .41, *p* < .001). When examining the correlation between delirium nursing care performance and each dimension of patient safety culture, significant positive correlations were found between delirium nursing care performance and unit/work area (r = .38, *p* < .001), immediate supervisor/manager (r = .18, *p* = .008), communication (r = .28, *p* < .001), reporting of patient safety events (r = .31, *p* < .001), hospital (r = .32, *p* < .001), patient safety knowledge/attitude (r = .34, *p* < .001), patient safety policy/procedure (r = .41, *p* < .001), and patient safety priority (r = .22, *p* = .002) (Table 3).

**Table 3:** Correlations among patient safety culture and nursing care of delirium (N = 214)

Variables	1	2	3	4	5	6	7	8	9	10
	r(*p*)	r(*p*)	r(*p*)	r(*p*)	r(*p*)	r(*p*)	r(*p*)	r(*p*)	r(*p*)	r(*p*)
1	1									
2	0.88(< 0.001)	1								
3	0.75(< 0.001)	0.62(< 0.001)	1							
4	0.82(< 0.001)	0.59(< 0.001)	0.64(< 0.001)	1						
5	0.47(< 0.001)	0.30(< 0.001)	0.20(0.003)	0.39(< 0.001)	1					
6	0.87(< 0.001)	0.73(< 0.001)	0.63(< 0.001)	0.63(< 0.001)	0.34(< 0.001)	1				
7	0.71(< 0.001)	0.49(< 0.001)	0.52(< 0.001)	0.61(< 0.001)	0.36(< 0.001)	0.54(< 0.001)	1			
8	0.76(< 0.001)	0.60(< 0.001)	0.46(< 0.001)	0.52(< 0.001)	0.42(< 0.001)	0.69(< 0.001)	0.54(< 0.001)	1		
9	0.67(< 0.001)	0.61(< 0.001)	0.52(< 0.001)	0.48(< 0.001)	0.15(0.029)	0.60(< 0.001)	0.34(< 0.001)	0.36(< 0.001)	1	
10	0.41(< 0.001)	0.38(< 0.001)	0.18(0.008)	0.28(< 0.001)	0.31(< 0.001)	0.32(< 0.001)	0.34(< 0.001)	0.41(< 0.001)	0.22(0.002)	1

(1) Patient safety culture; (2) Unit/Work area; (3) Supervisor, Manager, or Clinical leader; (4) Communication; (5) Reporting patient safety events; (6) Hospital; (7) Patient safety knowledge and attitude; (8) Patient safety policy and procedure; (9) Patient safety priority; (10) Nursing care for delirium

### Factors influencing nursing care for delirium

The factors influencing delirium nursing care among LCF nurses were found to be patient safety cultures (β = 0.34, *p* < .001), the presence of manuals for delirium in the ward (β = 0.25, *p* < .001), education on delirium (β = 0.23, *p =* .001), need for education on delirium (β = 0.17, *p =* .003), nurse-to-patient ratio (β=-0.13, *p =* .020), and marital status (β = 0.12, *p =* .031) (Table 4).

**Table 4:** Factors influencing nursing care for delirium (N = 214)

	Variables	Model 1				Model 2			Model 3		
		B	β(*p*)		t	B	β(*p*)	t	B	β(*p*)	t
Individual	Marital status (ref.=Married)	0.15	0.14(0.040)		2.07	0.19	0.18(0.003)	3.05	0.13	0.12(0.031)	2.17
Organizational	Nurse-to-patient ratio (ref.=≥30 )					− 0.10	− 0.11(0.060)	-1.89	− 0.12	− 0.13(0.020)	-2.35
Focus on delirium	Experience of education on delirium (ref.=Yes)								0.24	0.23(0.001)	3.49
	Pathway to nursing education on delirium (ref.=at the college)								0.02	0.02(0.759)	0.31
	Presence of manuals for delirium care in the ward (ref.=Yes)								0.25	0.25(< 0.001)	4.24
	Necessity of education on delirium (ref.=Yes)								0.50	0.17(0.003)	3.05
	Patient safety rating (ref.=Very good)								0.06	0.03(0.314)	1.01
Patient safety culture	Patient safety culture								0.33	0.34(< 0.001)	5.59
F(*p*)		4.29(0.040)		13.93(< 0.001)				17.88(< 0.001)			
R^2^		0.02		0.32				0.41			
Adjusted R^2^		0.02		0.30				0.39			

## Discussion

This study identified the factors associated with patient safety culture on delirium nursing care performance among nurses in 12 LCFs across three cities. Patient safety culture among LCF nurses showed an average score of 3.47 out of 5. This was comparable to an average of 3.37 reported in a previous study [21] analyzing patient safety culture among nurses in general wards, outpatient departments, and special wards in general hospitals (although a different measurement tool was used). However, it was lower than the 3.61 for nurses in university hospitals and general hospitals with integrated nursing and caregiving service wards, [22] and higher than the 3.28 for nurses in tertiary general hospitals [23]. These findings indicate that there are differences in perceptions of patient safety culture among hospitals and departments within hospitals.

The performance of delirium nursing care of LCF nurses scored an average of 2.75 points out of 4, which was lower than the 2.95 reported by hospice delirium care nurses in a previous study using the same tool [24]. The marginally lower delirium care performance among LCF nurses may be because patients admitted to LCFs often have multiple symptoms associated with their diseases, such as dementia, depression, and Parkinson’s disease. Differentiating the symptoms of various diseases from those of delirium can be challenging [25, 26] and may have contributed to the lower delirium care performance score observed in this study. Further, hypoactive delirium, a subtype of delirium, commonly occurs in elderly patients admitted to LCFs. The symptoms of hypoactive delirium including slowed speech, reduced mobility, apathy, depression, and lethargy, are often difficult for healthcare professionals to detect, [12, 25] making it hard to assess and intervene appropriately. Therefore, to enhance the delirium care performance of LCF nurses, more specialized education must be provided, focusing particularly on distinguishing delirium from other conditions, including behavioral symptoms associated with dementia in which early differentiation is challenging [15, 26]. Specialized education and awareness of hypoactive delirium are especially crucial.

The correlation analysis between patient safety cultures and delirium nursing care performance among LCF nurses in this study revealed significant correlations. These included unit/work area, immediate supervisor/manager, communication, reporting of patient safety events, hospital, patient safety knowledge/attitude, patient safety policy/procedure, and patient safety priority. Although research targeting LCF nurses is lacking for comparison purposes, a higher patient safety culture can be inferred to lead to improved performance of delirium care among LCF nurses based on the correlation found between patient safety culture and patient safety nursing activities (including partial delirium care activities) [27].

Hierarchical regression analysis was conducted to examine the influence of patient safety culture on delirium nursing care performance among LCF nurses. The result showed that factors influencing delirium care included marital status, nurse-to-patient ratio, experience of delirium care education, the presence of manuals for delirium care in wards, delirium care education, and patient safety culture.

First, being married was found to be associated with higher delirium nursing care performance, compared with being unmarried, which aligns with the results of a previous study on nurses in general hospitals [15]. Additionally, factors such as the nurse-to-patient ratio, delirium care education, presence of manuals for delirium care in wards, and the necessity of delirium care education were found to influence delirium care performance.

Delirium care performance was found to be lower when the nurse-to-patient ratio exceeded 30 patients per nurse. This is consistent with research [28] that reported negative impacts on patient outcomes, such as increased mortality and longer hospital stays, with an increasing number of patients assigned to a single nurse. Moreover, considering that the level of nurse staffing has an influence on patient safety outcomes and that the nurse-to-patient ratio is associated with positive or negative patient safety outcomes, [29] organizational support for staffing is necessary to enhance the delirium care performance of LCF nurses.

Delirium care education, the presence of manuals for delirium care in wards, and the delirium education were identified as factors affecting delirium nursing performance. This highlights the importance of education on delirium in the elderly, even in LCFs where awareness of delirium may be low and systematic delirium care may not be fully implemented [13]. This finding is consistent with a previous study [15] involving general hospital nurses, which also identified delirium care education as a key factor influencing delirium care. Another study [20] supports the finding that delirium care guidelines improve delirium care. Thus, developing tailored delirium care guidelines for patients in LCFs is crucial, where the majority of patients are at high risk for delirium with high incidence rates. This is supported by a study in general hospitals that reported significant correlations between delirium-related knowledge, nursing confidence, and delirium care performance [30].

Second, higher levels of patient safety culture were associated with higher delirium care performance. Although there are minimal studies with which to directly compare the findings, previous research [27] identified patient safety culture as a factor influencing patient safety nursing activities, including elements of delirium care. Further, it is necessary to consider factors such as management, administration, communication, and hospital policies, which are subdomains of patient safety culture, [6] to promote more proactive delirium care. If delirium care can become more effective by considering these factors, they will ultimately contribute to improving patient safety and nursing quality.

In this study, a dimension of patient safety culture related to the reporting of patient safety events scored the lowest, with 3.13. This was lower than the 3.61 reported by nurses in tertiary general hospitals [23]. This difference may be because reporting patient safety events is not a pleasant experience for anyone, and unless there is a culture of actively promoting and encouraging such reporting, the reporting rate will inevitably be low. Moreover, blaming, guilt, and punitive cultures toward employees who report patient safety events hinder the proper handling and prevention of such events [31]. Therefore, it is necessary to promote a non-punitive culture and develop reporting systems that are suitable for LCF operations to facilitate more proactive reporting of patient safety events.

### Implications

This study is significant in that it conducted research on 12 LCFs in three cities, analyzing the extent of delirium care and influencing factors of patient safety cultures with a diverse range of LCF nurses in Korea. Additionally, because of the lack of patient safety culture tools suitable for domestic LCFs, this study made efforts to modify and supplement AHRQ’s HSOPSC Version 2.0^19^ and the Korean Patient Safety Culture tool [4] to reflect the environment and characteristics of domestic LCFs as accurately as possible. The results indicate that tailored delirium nursing education is necessary to enhance delirium care in LCFs and reinforce patient safety culture as it confirmed that patient safety culture influences delirium nursing care performance.

### Limitations

A limitation of this study is that there were no survey items regarding delirium nursing care or patient safety culture amid the pandemic despite the prevalence of COVID-19 at the time of the survey. Consequently, the influence of COVID-19 on the practice of delirium care by LCF nurses was not considered. Further, this study was limited in that the delirium nursing care tool used in this study was not specifically designed for LCF nurses, but for nurses providing delirium care for cancer patients [20]. Therefore, future research should focus on developing tools for measuring delirium nursing care tailored to LCFs.

## Conclusion

The factors influencing delirium nursing care by LCF nurses identified in this study include nurse-to-patient ratio, education on delirium care, and patient safety culture. Tailored delirium education and guidelines are necessary for LCF nurses who focus on elderly patients to enhance their delirium nursing care performance. Additionally, policies to reduce the patient load per nurse are necessary. It is important to develop and incorporate tailored delirium education and guidelines for LCF residents into a program that strengthens the patient safety culture suitable for the characteristics of LCFs.

## Data Availability

The datasets used and/or analysed during the current study are available from the corresponding author on reasonable request.

## List of abbreviations

Long: term care facilities (LCF)

## References

[1] World Health Organization. Patient safety. Accessed August 31., 2021. https://www.who.int/health-topics/patient-safety#tab=tab_2.

[2] Bartonickova D, Kalankova D, Ziakova K. How to measure patient safety culture a literature review of instruments. Acta Med Martiniana. 2021;21(2). 10.2478/acm-2021-0010.

[3] Rockville W, Sorra J, Yount N, Famolaro T, Gray L (2021). Hospital survey on patient safety culture version 2.0: user’s guide.

[4] Lee SG (2015). Development and psychometric evaluation of the Korean patient Safety Culture Survey Instrument for hospitals [doctoral degree].

[5] Institute for Healthcare Improvement. (2014, 2022 April 23). Develop a culture of safety. http://www.ihi.org/resources/Pages/Changes/DevelopaCultureofSafety.aspx.

[6] Lee JS, Lee SY (2021). Ethical climate and patient safety competencies between nurses in long-term care hospital. J Convergence Cult Technol.

[7] Park SU, Yeom EY (2016). Experience of the role conflict of nurses in long-term care hospitals. Int J Contents.

[8] Ko YK, Park BH (2014). The relationship of the nursing work environment and nursing outcome among nurses and content analysis of nurses` workload. Korean Soc Hosp Manage.

[9] Kim SM, Jeong SH, Lee MH, Kim HK, Importance (2017). Performance and rates of nurse performance of nursing interventions in long-term care hospitals. J Korean Acad Nurs Adm.

[10] Hessels A, Paliwal M, Weaver SH, Siddiqui D, Wurmser TA (2019). Impact of patient safety culture on missed nursing care and adverse patient events. J Nurs Care Qual.

[11] American Psychiatric Association (2013). Diagnostic and statistical manual of mental disorders DSM-V.

[12] Oh ES, Fong TG, Hshieh TT, Inouye SK (2017). Delirium in older persons: advances in diagnosis and treatment. JAMA.

[13] Moon KJ, Park HO (2018). Outcomes of patients with delirium in long-term care facilities: a prospective cohort study. J Gerontol Nurs.

[14] Lee J (2020). Risk factors for nursing home delirium a systematic review. J Korean Gerontol Nurs.

[15] Kang JS, Song HJ (2019). Factors affecting the performance of nurses in delirium care. J Korean Crit Care Nurs.

[16] Kim SM, Jeong SH, Lee MH, Kim HK (2017). Importance, performance and rates of nurse performance of nursing interventions in long-term care hospitals. J Korean Academy Nurs Admin.

[17] Jeong SY, Lee UK (2019). Factors associated with compassion satisfaction of nurses in long-term care hospitals: focused on patient safety culture. Korean Soc Public Health Nurs.

[18] Lee SE, Dahinten VS (2021). Adaptation and validation of a korean-language version of the revised hospital survey on patient safety culture (K-HSOPSC 2.0). BMC Nurs.

[19] Agency for Healthcare Research and Quality. Hospital survey on patient safety culture version 2.0. 2019 Accessed December 2, 2021. https://www.ahrq.gov/sites/default/files/wysiwyg/sops/surveys/hospital/SOPS-Hospital-Survey-2.05-26-2021.pdf.

[20] Park YS, Gu MO (2013). The development and effects of evidence-based nursing practice guideline for cancer patients with Delirium. Korean Soc Evid Based Nurs.

[21] Kim SA, Kim EM, Lee JR, Oh EG (2018). Effect of nurses’ perception of patient safety culture on reporting of patient safety events. J Korean Acad Nurs Admin.

[22] Choi HJ, Lee YM, Park HJ. Effects of awareness of patient safety culture, emotional labor and job stress on patient safety nursing activities by comprehensive nursing care medical service ward nurses. J Korean Critical Care Nurs. 2021;14(3). 10.34250/jkccn.2021.14.3.87.

[23] Park JH (2020). Effects of nurses’ patient safety management importance, patient safety culture and nursing service quality on patient safety management activities in tertiary hospitals. J Korean Acad Nurs Admin.

[24] Jang BJ, Yeom HA (2018). Hospice-palliative care nurses’ knowledge of delirium, self-efficacy and nursing performance on delirium. Korean J Hosp Palliat Care.

[25] National Institute for Health and Care Excellence. (2018, 2022 June 3). 2018 Surveillance of delirium prevention, diagnosis and management (NICE Guideline CG103). https://www.nice.org.uk/guidance/cg103/resources/2018-surveillance-of-delirium-prevention-diagnosis-and-management-nice-guideline-cg103-pdf-8546233843141. 31846262

[26] Park M, Ja Moon K (2023). Web-based delirium application for long-term care facilities. J Am Med Dir Assoc.

[27] Noh S, Kim TI (2021). The effects of organizational commitment and perceived patient safety culture on patient safety nursing activities among nurses in comprehensive nursing care units. Hosp Nurses Assoc.

[28] Lasater KB, Aiken LH, Sloane D, French R, Martin B, Alexander M, McHugh MD (2021). Patient outcomes and cost savings associated with hospital safe nurse staffing legislation: an observational study. BMJ Open.

[29] Carthon JMB, Davis L, Dierkes A, Hatfield L, Hedgeland T, Holland S, Aiken LH (2019). Association of nurse engagement and nurse staffing on patient safety. J Nurs Care Qual.

[30] Park HM, Chang MY (2016). Nurse’s knowledge, confidence and performance on care for delirium. J Health Info Stat.

[31] Lee SE, Scott LD, Dahinten VS, Vincent C, Lopez KD, Park CG (2019). Safety culture, patient safety, and quality of care outcomes: a literature review. West J Nurs Res.

